# Individual surveillance by competing risk model for patients with hepatocellular carcinoma occurrence in all-cause cirrhosis

**DOI:** 10.1007/s00432-023-04911-y

**Published:** 2023-07-26

**Authors:** Qi Wang, Dandan Guo, Wenfeng Gao, Chunwang Yuan, Jianjun Li, Yinghua Zhang, Ning He, Peng Zhao, Jiasheng Zheng, Yonghong Zhang

**Affiliations:** 1 Research Center for Biomedical Resources, Beijing You’an Hospital, Capital Medical University, Beijing, 100069 China; 2 Interventional Therapy Center for Oncology, Beijing You’an Hospital, Capital Medical University, Beijing, 100069 China

**Keywords:** Cirrhosis, Hepatocellular carcinoma, Predict, Competing risk model, Prospective

## Abstract

**Purpose:**

It was of great significance to identify someone with a high risk of hepatocellular carcinoma (HCC) occurrence and make a diagnosis as early as possible. Therefore, we aimed to develop and validate a new, objective, and accurate prediction model, and convert it into a nomogram to make a personalized prediction of cancer occurrence in cirrhotic patients with different etiologies.

**Methods:**

The present study included 938 patients with cirrhosis from January 1, 2011, to December 31, 2012. Patients were prospectively followed-up until January 1, 2018. We used a competing risk model and the Fine–Gray test to develop and validate the prediction model and to plot a nomogram based on the model established.

**Results:**

At the end of follow-up, 202 (21.5%) patients developed HCC, with a 5-year incidence of 19.0% (corrected for competing risk model). Based on the competing risk regression method, we built a prediction model including age, gender, etiology, lymphocyte, and A/G ratio. Three groups with different risks were generated on account of tertiles of the 5-year risk predicted by the model. The cumulative 1-, 3-, and 5-year incidences of HCC were 2.0%, 20.8%, and 42.3% in high-risk group, 0.9%, 10.1%, and 17.7% in medium-risk group, and 0%, 2.0%, 8.5% in low-risk group (*P* < 0.001). The model showed excellent discrimination and calibration in predicting the risk of HCC occurrence in patients with all-cause cirrhosis.

**Conclusion:**

The model could make an individual prediction of cancer occurrence and stratify patients based on predicted risk, regardless of the causes of cirrhosis.

**Supplementary Information:**

The online version contains supplementary material available at 10.1007/s00432-023-04911-y.

## Implications for practice

To improve long-term survival in patients with HCC, it is urgent to identify individuals with high risk of cancer occurrence and make a diagnosis as early as possible. Existing models are established based on Cox proportional hazards model, and these studies do not take competing risk events into account. Using the competing risk model, we developed and validate a new prediction tool. Our model could screen out patients with low risk of cancer occurrence, who could receive less intensive cancer surveillance. In contrast, for patients with the high cancer risk, enhanced follow-up was recommended for screening and diagnosis of HCC.

## Introduction

Hepatocellular carcinoma (HCC) is one of the leading causes of tumor-related death worldwide (Sung et al. 2021). The prognosis of patients with different tumor stages varies significantly, with a 5-year overall survival (OS) rate of 70–75% in the early stage, while with an average survival time of less than 12 months in the advanced stage (Llovet and Bruix 2000; Ioannou et al. 2008; Villanueva 2019). It is reported that the 5-year OS rate of liver cancer in China is only about 12.1% (Zeng et al. 2018). This is mainly due to the low rate of early diagnosis, that is, most patients have advanced tumors when they are diagnosed (Trinchet et al. 2011). As a routine monitoring technique, ultrasound has poor sensitivity for small tumors (< 2 cm), which can be accurately diagnosed by contrast-enhanced computed tomography (CT) or magnetic resonance imaging (MRI) (Singal et al. 2014). However, these expensive imaging technologies are not cost-effective for someone with cirrhosis who are at low risk of cancer occurrence (Kim et al. 2017; Cadier et al. 2017).

To improve long-term survival in patients with HCC, it is urgent to identify individuals with a high risk of cancer occurrence and make a diagnosis as early as possible. So far, several models such as PAGE-B, mPAGE-B, REAL-B, THRI, and so on have been developed and validated to assess the occurrence risk of liver cancer (Yang et al. 2020; Kim et al. 2018; Hu et al. 2020; Papatheodoridis et al. 2016; Ioannou et al. 2018, 2019; Yu et al. 2019; Sharma et al. 2017). The cases of non-viral-related liver diseases like alcoholic liver disease (ALD) and non-alcoholic fatty liver disease (NAFLD) are increasing year by year, which is endangering human life and health. However, most of these models are based on selected patients with hepatitis B virus- (HBV) or hepatitis C virus- (HCV) associated liver disease, with a condition of antiviral treatment. Therefore, the generalization of the models in patients with all-cause cirrhosis is somewhat limited (Heimbach et al. 2018). From the perspective of statistical methods, all these models are established based on Cox proportional hazards model, and these studies do not take competing risk events into account. For Cox proportional hazards model, death is treated as a censoring event, that is to say, it is believed that cancer occurrence will be observed in the case of continued follow-up. Whereas this is not the case, and Cox regression often overestimates the cumulative incidence risk (Putter et al. 2007; Berry et al. 2010).

In conclusion, using the competing risk model, we aimed to develop and validate a new prediction tool that was more consistent with the real-world association model, and convert it into a nomogram to make an individualized prediction of cancer risk in patients with all-cause cirrhosis in this large and prospective cohort study.

## Methods

### Patients enrolled

A total of 938 patients with all-cause cirrhosis who were admitted to Beijing You’an Hospital affiliated with Capital Medical University from January 1, 2011, to December 31, 2012, were enrolled. All patients were diagnosed with cirrhosis by imaging and histological examination based on etiology, medical history, clinical manifestation, and complications. Generally speaking, splits by different hospitals or by admission time were both attractive approaches to develop and externally validate a prediction model, whereas our study divided the data set into derivation cohort and external validation cohorts by the time of visit (known as temporal validation) (Moons et al. 2012; Steyerberg and Harrell 2016). Therefore, to improve the generalization capability and external applicability of the model, 457 patients treated in 2011 were included in the derivation cohort, and 481 patients treated in 2012 were included in the validation cohort for temporal validation.

### Clinicopathological data

Thirty-four indicators, including demographic and baseline clinicopathological data, were collected and summarized as follows: (1) demographic data: age, gender, the history of antiviral, hypertension and diabetes mellitus; (2) etiology of cirrhosis: HBV, HCV, ALD, co-infection, and others (NAFLD, primary biliary cirrhosis, autoimmune hepatitis, drug-induced liver injury, Budd–Chiari syndrome, etc.); (3) blood routine examination: white blood cell, neutrophil, lymphocyte, monocyte, hemoglobin, platelet; (4) liver and renal function examination: Child–Pugh class; alanine aminotransferase (ALT), aspartate aminotransferase (AST), total bilirubin (TBIL), direct bilirubin (DBIL), total protein (TP), albumin, globulin, γ-glutamyl transpeptidase (γ-GT), alkaline phosphatase (ALP), prealbumin, total bile acid (TBA), cholinesterase (ChE), cholesterol; (5) coagulation markers: prothrombin time (PT), prothrombin time activity (PTA), international normalized ratio (INR), fibrinogen, activated partial thromboplastin time (APTT), thrombin time (TT); (6) other indicators: alpha fetoprotein (AFP) and viral load.

Compared with albumin or globulin alone, the albumin-to-globulin ratio (A/G ratio) is not easily affected by changes in body fluids, such as hemoconcentration or hemodilution. In modeling, therefore, we included the A/G ratio, which can be used as a more objective and stable clinical parameter to assess the risk of cancer occurrence in patients with cirrhosis.

### Follow-up

The enrolled patients in both the derivation and validation cohorts were followed-up every 6 months in the outpatient clinic, including medical examination, laboratory, and ultrasonic examination. Once focal lesions were reported, contrast-enhanced CT or MRI and/or histological examination were performed immediately for definitive diagnosis in accordance with the diagnostic procedures recommended by the AALSD guidelines (Heimbach et al. 2018). Therefore, follow-up strategies were consistent between the derivation and validation cohorts. Events (e.g., death, cause of death, occurrence of hepatocellular carcinoma, liver transplantation) during follow-up were recorded in detail. The process of recording information was monitored by three Clinical Research Associates (LJJ, ZYH, and GWF). The medical diagnosis during the follow-up was confirmed by two senior hepatologists with 10 years of experience in our center (ZYH and ZJS).

### Standard of diagnosis

Diagnostic criteria for cirrhosis (one of the following three): (1) The presence of pseudolobule and regenerative nodule is reported on histological examination; (2) endoscopy demonstrates esophageal and gastric varices or ectopic varices, except for non-cirrhotic portal hypertension; (3) the results of ultrasonography, liver stiffness measurement (LSM) or CT suggest the characteristics of cirrhosis or portal hypertension, such as splenomegaly, ascites, hepatic encephalopathy, portal vein diameter not less than 1.3 cm (Ginès et al. 2021). For nodules > 1 cm detected by ultrasound examination, contrast-enhanced CT or MRI is performed. Diagnosis of HCC is made when at least one imaging examination showed significant enhancement in the arterial phase while washout in the portal vein phase and/or delayed phase. For patients with atypical imaging features but suspected malignant nodules reported by CT or MRI, a further needle biopsy is required to confirm the diagnosis (Heimbach et al. 2018).

### Statistical analyses

Logarithmic transformation was performed for continuous data that did not conform to the normal distribution. If the data after transformation still did not conform to the normal distribution, the original data would be retained and appropriate statistical methods were selected for analysis. The continuous data conforming to the normal distribution were represented by the means ± standard deviation, and if not, by the median (interquartile range, IQR). Categorical data were expressed as frequency or percentage. Depending on whether the data obeyed the normal distribution, Student's *t *test or Mann–Whitney *U* test was used between the two groups, and a one-way ANOVA analysis or Kruskal–Wallis test was performed between the three groups for continuous data. The Chi-square test was used for the different comparison of categorical data. The non-linear relationship between variables and outcome was analyzed using the restricted cubic spline (RCS) method with five knots. The cumulative incidence curve was plotted to assess the time-dependent cumulative incidences of primary endpoint and competing risk events.

In this study, death or liver transplantation (represented by the number 2) would hinder cancer occurrence (represented by the number 1), and there was competing risk between 1 and 2, which were mutually competing risk events. Therefore, a competing risk model was used to screen independent risk factors and establish the model. Variables with a *P* value less than 0.1 in univariate analysis were included in multivariate analysis. Sub-hazard ratios (SHRs) and 95% confidence interval (CI) were reported, with regression coefficients (log [SHR]) considered as weights to calculate the predicted risk score and plot the nomogram. After the model was established, internal and external validation was carried out based on discrimination, calibration, and clinical value. The Bootstrap sampling method was performed to calculate Harrell's concordance index. Calibration curves were drawn to evaluate the degree of consistency between the predicted and the observed probability. Three groups with different occurrence risks (low-risk, medium-risk, and high-risk) were generated on account of tertiles of the 5-year risk predicted by the model established. The cumulative incidence curves of the three groups were plotted for clinical applicability analysis.

It was difficult to calculate the sample size beforehand due to weak evidence in establishing a risk stratification model for predicting the development of HCC in cirrhotic patients. Nevertheless, the high number of HCC incidences (more than 200) compared with the number of Cox model variables (5) implied that the “ten events per variable” rule was largely exceeded, thus indicating sufficient accuracy and precision of estimates (Peduzzi et al. 1995).

A *P* value less than 0.05 was considered statistically significant. All statistical analyses were conducted by R software version 4.0.5 (R Foundation for Statistical Computing, Vienna, Austria).

## Results

### Baseline characteristics of patients enrolled

A total of 938 patients with all-cause cirrhosis admitted to our hospital from January 1, 2011, to December 31, 2012, were included, with an average age of 51 years. There were 602 cases of HBV, 109 of HCV, 104 of ALD, 9 of HBV/HCV co-infection, and 114 of other etiologies (2 NAFLD, 27 primary biliary cirrhosis, 12 autoimmune hepatitis, 10 drug-induced liver injury, 2 Budd–Chiari syndromes, and 61cryptogenic cirrhosis). There were 645 cases (68.8%) in males, 156 (16.6%) with hypertension, 137 (14.6%) with diabetes mellitus, and 617 (65.8%) with antiviral history. 453 (48.3%) patients were Child–Pugh class A, 308 (32.8%) were class B, and 177 (18.9%) were class C.

We divided patients into three groups (cirrhosis group, cancer group, and competing event group) based on patient status during follow-up, and compared the baseline data of the three groups (Table 1 and Supplementary Fig. 1). Interestingly, among the 34 indicators studied by us, 31 had significant statistical differences (P < 0.05), and the *P* values of the remaining 3 were critically positive (*P* = 0.05–0.1). The baseline data of the derivation and validation cohort were compared (Table 2). The results showed that in addition to differences in some parameters (ALT, AST, TBA, etc.), the number of patients with HCC occurrence was higher in the derivation cohort (26.0% versus 17.3%).

**Table 1:** Comparison of clinical data between the groups of cirrhosis, cancer, and competing risk events

Variables	Cirrhosis	Cancer	Competing risk events	*P* value
	*n* = 631	*n* = 202	*n* = 105	
Age (years old)	49.8 ± 10.3	54.3 ± 10.3	56.3 ± 12.1	** < 0.001**
Gender (male/%)	414 (65.6)	155 (76.7)	76 (72.4)	**0.009**
Hypertension (*n*/%)	93 (14.7)	39 (19.3)	24 (22.9)	0.061
Diabetes mellitus (*n*/%)	80 (12.7)	32 (15.8)	25 (23.8)	**0.010**
Antiviral history (*n*/%)	439 (69.6)	135 (66.8)	43 (41.0)	** < 0.001**
Etiology				** < 0.001**
Virus associated (*n*/%)	490 (77.7)	167 (82.6)	63 (60.0)	
Alcohol associated (*n*/%)	67 (10.6)	20 (10.0)	17 (16.2)	
Others (*n*/%)	74 (11.7)	15 (7.4)	25 (23.8)	
Child–Pugh class				** < 0.001**
A (*n*/%)	353 (55.9)	79 (39.1)	21 (20.0)	
B (*n*/%)	187 (29.6)	80 (39.6)	41 (39.0)	
C (*n*/%)	91 (14.5)	43 (21.3)	43 (41.0)	
ln(White blood cell, × 10^9/L)	1.36 ± 0.48	1.30 ± 0.45	1.48 ± 0.55	**0.011**
ln(Neutrophil, × 10^9/L)	0.83 ± 0.58	0.78 ± 0.57	1.00 ± 0.64	**0.006**
ln(Lymphocyte, × 10^9/L)	0.09 ± 0.53	0.02 ± 0.50	0.04 ± 0.61	**0.050**
ln(Monocyte, × 10^9/L)	– 1.39 ± 0.58	– 1.46 ± 0.54	– 1.25 ± 0.65	**0.012**
Hemoglobin (g/L)	124 (107.0–143.0)	121 (105.0–139.5)	111 (95.0–125.0)	** < 0.001**
ln(Platelet, × 10^9/L)	4.37 ± 0.57	4.22 ± 0.56	4.30 ± 0.54	**0.005**
Alanine aminotransferase (U/L)	37 (23–66)	43 (28–71.5)	36 (26–66)	0.085
Aspartate aminotransferase (U/L)	44 (31–72)	53 (37.3–80.0)	62 (36–94)	** < 0.001**
Total bilirubin (μmol/L)	24.1 (17.4–37.9)	28.8 (19.6–42.0)	35.1 (20.4–66.9)	** < 0.001**
Direct bilirubin (μmol/L)	5.1 (3.2–9.1)	6.6 (4.1–11.5)	10.1 (4.8–21.3)	** < 0.001**
Total protein (g/L)	68.3 (62.9–73.6)	67.4 (60.9–72.9)	65.5 (57.6–70.1)	** < 0.001**
Albumin (g/L)	37.7 ± 6.42	35.2 ± 6.42	32.7 ± 5.46	** < 0.001**
Globulin (g/L)	29.8 ± 5.99	31.3 ± 6.12	31.0 ± 7.48	**0.008**
Gamma-glutamyltransferase (U/L)	49 (27–95)	58 (31.3–108.5)	55 (27.0–113)	0.069
ln(Alkaline phosphatase,U/L)	4.48 ± 0.46	4.53 ± 0.38	4.60 ± 0.44	**0.035**
ln(Prealbumin, mg/L)	4.64 ± 0.54	4.48 ± 0.54	4.29 ± 0.50	** < 0.001**
ln(Total bile acid, μmol/L)	2.86 ± 1.24	3.08 ± 1.10	3.61 ± 1.03	** < 0.001**
ln(Cholinesterase, U/L)	8.37 ± 0.49	8.15 ± 0.55	8.02 ± 0.43	** < 0.001**
ln(Cholesterol, mmol/L)	1.28 ± 0.29	1.25 ± 0.29	1.16 ± 0.33	** < 0.001**
Prothrombin time (s)	13.8 (12.5–15.1)	14.6 (13.2–16)	14.9 (13.4–17.2)	** < 0.001**
Prothrombin time activity (%)	76 (68.3–86.1)	73 (63.3–82.3)	70 (60.1–77.5)	** < 0.001**
International normalized ratio	1.19 ± 0.20	1.23 ± 0.19	1.29 ± 0.22	** < 0.001**
ln(Fibrinogen, g/L)	0.60 ± 0.37	0.57 ± 0.39	0.49 ± 0.46	**0.019**
ln(APTT, sec.)	3.60 ± 0.21	3.63 ± 0.22	3.69 ± 0.23	** < 0.001**
Thrombin time (s)	19.8 ± 2.45	20.2 ± 2.29	20.8 ± 2.44	** < 0.001**
Alpha fetoprotein (positive/%)	191(30.3%)	94 (46.5%)	31 (29.5%)	** < 0.001**
Virus load (IU/mL)				**0.018**
< 1000	337 (53.4)	85 (42.1)	63 (60.0)	
1000–50,000	82 (13.0)	30 (14.9)	14 (13.3)	
> 50,000	212 (33.6)	87 (43.1)	28 (26.7)	

*APTT* activated partial thromboplastin time

**Table 2:** Comparison of clinical data between the groups of cirrhosis, cancer, and competing risk events

Variables	Total	Derivation cohort	Validation cohort	*P* value
	*n* = 938	*n* = 457	*n* = 481	
Outcomes				**0.003**
Cirrhosis (*n*/%)	631 (67.3)	286 (62.6)	345 (71.7)	
Cancer (*n*/%)	202 (21.5)	119 (26.0)	83 (17.3)	
Death (*n*/%)	105 (11.2)	52 (11.4)	53 (11.0)	
Age (years old)	51.5 ± 10.8	52.2 ± 10.7	50.8 ± 10.8	0.059
Gender (male/%)	645 (68.8)	326 (71.3)	319 (66.3)	0.113
Hypertension (*n*/%)	156 (16.6)	73 (16.0)	83 (17.3)	0.660
Diabetes mellitus (*n*/%)	137 (14.6)	68 (14.9)	69 (14.3)	0.889
Antiviral history (*n*/%)	617 (65.8)	299 (65.4)	318 (66.1)	0.879
Etiology				0.843
Virus associated (*n*/%)	720 (76.8)	354 (77.5)	366 (76.1)	
Alcohol associated (*n*/%)	104 (11.0)	48 (10.5)	56 (11.6)	
Others (*n*/%)	114 (12.2)	55 (12.0)	59 (12.3)	
Child–Pugh class				0.729
A (*n*/%)	453 (48.3)	218 (47.7)	235 (48.8)	
B (*n*/%)	308 (32.8)	148 (32.4)	160 (33.3)	
C (*n*/%)	177 (18.9)	91 (19.9)	86 (17.9)	
ln(White blood cell, × 10^9/L)	1.36 ± 0.49	1.35 ± 0.49	1.37 ± 0.49	0.475
ln(Neutrophil, × 10^9/L)	0.84 ± 0.58	0.83 ± 0.59	0.85 ± 0.58	0.528
ln(Lymphocyte, × 10^9/L)	0.07 ± 0.53	0.07 ± 0.052	0.07 ± 0.054	0.979
ln(Monocyte, × 10^9/L)	– 1.39 ± 0.58	– 1.45 ± 0.59	– 1.33 ± 0.57	**0.003**
Hemoglobin (g/L)	122 (105.0–141.0)	122 (105.0–142.0)	121 (104.0–139.0)	0.554
ln(Platelet, × 10^9/L)	4.33 ± 0.56	4.34 ± 0.58	4.32 ± 0.55	0.563
Alanine aminotransferase, U/L	38 (24–67)	43 (27–75)	34 (22–61)	** < 0.001**
Aspartate aminotransferase (U/L)	47 (32–79)	51 (34–83)	45 (31–72)	** < 0.001**
Total bilirubin (μmol/L)	25.5 (17.9–41.4)	25.5 (18.2–44.9)	25.5 (17.7–39.9)	0.211
Direct bilirubin (μmol/L)	5.5 (3.5–11.2)	5.8 (3.6–12.2)	5.4 (3.5–10.6)	0.249
Total protein (g/L)	67.8 (61.7–73.2)	67.6 (61.0–73.3)	67.9 (62.5–73.2)	0.582
Albumin (g/L)	36.6 ± 6.53	36.5 ± 6.55	36.7 ± 6.52	0.584
Globulin (g/L)	30.3 ± 6.22	30.2 ± 6.35	30.3 ± 6.11	0.778
Gamma-glutamyltransferase (U/L)	51 (28.0–101.0)	51 (29–106)	51 (28.0–98.0)	0.478
ln(Alkaline phosphatase, U/L)	4.51 ± 0.44	4.53 ± 0.43	4.49 ± 0.45	0.179
ln(Prealbumin, mg/L)	4.57 ± 0.55	4.63 ± 0.52	4.51 ± 0.56	** < 0.001**
ln(Total bile acid, μmol/L)	2.99 ± 1.21	3.07 ± 1.19	2.91 ± 1.22	**0.044**
ln(Cholinesterase, U/L)	8.29 ± 0.51	8.28 ± 0.52	8.29 ± 0.51	0.751
ln(Cholesterol, mmol/L)	1.26 ± 0.30	1.27 ± 0.30	1.25 ± 0.30	0.218
Prothrombin time (s)	14.2 (12.7–15.5)	14.6 (12.8–16.3)	13.8 (12.6–15.0)	** < 0.001**
Prothrombin time activity (%)	74.9 (65.8–84.0)	74.9 (63.0–84.1)	75.3 (68.3–84.0)	**0.016**
International normalized ratio	1.21 ± 0.20	1.23 ± 0.22	1.19 ± 0.18	** < 0.001**
ln(Fibrinogen, g/L)	0.58 ± 0.39	0.59 ± 0.39	0.58 ± 0.39	0.492
ln(APTT, s)	3.62 ± 0.21	3.59 ± 0.21	3.64 ± 0.22	** < 0.001**
Thrombin time (s)	20.0 ± 2.43	20.3 ± 2.45	19.8 ± 2.40	**0.005**
Alpha fetoprotein (positive/%)	316 (33.7)	173 (37.8)	143 (29.7)	**0.010**
Virus load (IU/mL)				0.106
< 1000	485 (51.7)	221 (48.4)	264 (54.9)	
1000–50,000	126 (13.4)	69 (15.1)	57 (11.9)	
> 50,000	327 (34.9)	167 (36.5)	160 (33.3)	

*APTT* activated partial thromboplastin time

### Follow-up and patients outcomes

Sixty-eight cases were lost to follow-up with the last follow-up time of January 1, 2018. The median follow-up time was 66.2 months (IQR: 48.4–74.4). Sixteen patients received liver transplantation. By the end of the follow-up, 202 patients developed HCC. The etiologies of 202 HCC patients were summarized as follows: 138 cases of HBV, 24 cases of HCV, 20 cases of ALD, 15 cases of other causes, and 5 cases of co-infection. The cumulative cancer incidences of 1, 3, and 5 years were 1.5% (14/938), 10.1% (95/938), 18.1% (170/938), and 5-year incidence corrected by the competing risk model was 19.0% (Supplementary Fig. 2). The characteristics of HCC are summarized in Supplementary Table 1. When diagnosed, about 70% of patients had single or small tumors, and about 65% had BCLC stage A.

**Fig. 1:**
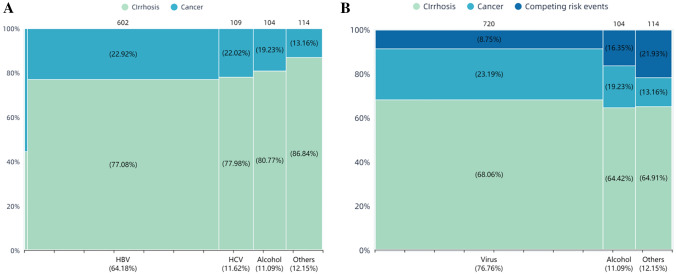
Marimekko plot for analyzing incidence of hepatocellular carcinoma and mortality in patients with cirrhosis of different etiologies. *HBV* hepatitis B virus, *HCV* hepatitis C virus

A total of 89 (9.5%) patients died, with a 5-year OS of 92.7%. The causes of death included gastrointestinal bleeding in 27 (30.3%) cases, spontaneous bacterial peritonitis in 23 (25.8%) cases, liver failure in 18 (20.2%), hepatic encephalopathy in 11 (12.4%) cases, multiple organ failure in 4 (4.5%) cases, and others causes in 6 (6.8%) cases (2 hepatorenal syndromes, 2 pulmonary infections, 2 other malignant diseases (1 colorectal cancer, 1 gastric cancer)). Marimekko plots were used to analyze the occurrence of cancer and death among patients with various etiologies and found that patients with virus-associated cirrhosis had the highest incidence of cancer (23.19%) and the lowest mortality (8.75%). Five (55.6%) of nine patients with co-infection had HCC (Fig. 1a). Notably, patients with cirrhosis of other etiologies had the highest mortality (21.93%) and the lowest cancer incidence (13.16%) (Fig. 1b).

### Competing risk model for predicting HCC occurrence

The unadjusted univariate analysis and multivariate competing risk regression were performed (Table 3). Univariate analysis showed that 14 indicators including age, gender, etiology, Child–Pugh class, lymphocyte, platelet, A/G ratio, prealbumin, ChE, PT, PTA, INR, AFP, and viral load were associated with increased occurrence risk of HCC. Five independent risk factors, involving male, old age, virus-associated cirrhosis, and low levels of lymphocyte and A/G ratio, were finally identified by multivariate competing risk regression analysis and then incorporated into the model (YOUAN model). Although the *P* value of lymphocyte was 0.077 in multivariate analysis, it was well known that low lymphocyte was related to poor prognosis of many diseases. Therefore, to improve the performance, lymphocyte was considered in the model, which increased Harrell's concordance index from 0.718 to 0.732.

**Table 3:** Predictors of hepatocellular carcinoma occurrence based on competing risk model

Variables	Univariate		Multivariate		
	SHR (95% CI)	*P* value	aSHR(95%CI)	coefficient	*P* value
Age (years old)	1.03 (1.01–1.05)	** < 0.001**	1.03 (1.02–1.05)	0.034	** < 0.001**
Gender (male/%)	0.64 (0.42–0.99)	**0.046**	0.50 (0.33–0.77)	– 0.692	**0.001**
Hypertension (*n*/%)	0.86 (0.52–1.44)	0.560			
Diabetes mellitus (*n*/%)	1.11 (0.67–1.82)	0.690			
Antiviral history (*n*/%)	1.17 (0.79–1.73)	0.440			
Etiology	0.71 (0.50–1.01)	**0.054**	0.61 (0.43–0.86)	– 0.498	**0.006**
Child–Pugh class	1.26 (1.02–1.57)	**0.035**	1.03 (0.69–1.53)	0.025	0.900
ln(White blood cell, × 10^9/L)	0.75 (0.51–1.09)	0.130			
ln(Neutrophil, × 10^9/L)	0.87 (0.63–1.21)	0.410			
ln(Lymphocyte, × 10^9/L)	0.66 (0.47–0.93)	**0.017**	0.57 (0.39–0.84)	– 0.558	**0.077**
ln(Monocyte, × 10^9/L)	0.83 (0.62–1.11)	0.210			
Hemoglobin (g/L)	1.00 (0.99–1.01)	0.820			
ln(Platelet, × 10^9/L)	0.66 (0.48–0.93)	**0.015**	0.82 (0.52–1.28)	– 0.199	0.380
Alanine aminotransferase,U/L	1.00 (0.99–1.00)	0.350			
Aspartate aminotransferase (U/L)	1.00 (0.99–1.00)	0.460			
Total bilirubin (μmol/L)	1.0 (0.99–1.00)	0.990			
Direct bilirubin (μmol/L)	0.99 (0.99–1.00)	0.800			
Total protein (g/L)	1.03 (0.90–1.18)	0.680			
A/G ratio	0.45 (0.27–0.75)	**0.002**	0.40 (0.24–0.68)	– 0.918	** < 0.001**
Gamma-glutamyltransferase (U/L)	1.00 (0.99–1.00)	0.730			
ln(Alkaline phosphatase, U/L)	1.08 (0.75–1.54)	0.690			
ln(Prealbumin, mg/L)	0.73 (0.53–0.99)	**0.048**	1.29 (0.75–2.23)	0.255	0.360
ln(Total bile acid, μmol/L)	1.03 (0.90–1.18)	0.680			
ln(Cholinesterase, U/L)	0.58 (0.40–0.84)	**0.004**	0.78 (0.39–1.55)	– 0.250	0.480
ln(Cholesterol, mmol/L)	0.73 (0.41–1.29)	0.280			
Prothrombin time (sec.)	1.07 (1.01–1.13)	**0.025**	0.77 (0.52–1.15)	– 0.263	0.200
Prothrombin time activity (%)	0.99 (0.97–0.99)	**0.007**	0.98 (0.94–1.01)	– 0.025	0.210
International normalized ratio	2.32 (1.15–4.71)	**0.019**	8.64 (0.03–5.38)	2.16	0.450
ln(Fibrinogen, g/L)	0.77 (0.49–1.21)	0.260			
ln(APTT, sec.)	1.62 (0.65–4.08)	0.300			
Thrombin time (sec.)	1.02 (0.95–1.10)	0.540			
Alpha fetoprotein (positive/%)	1.54 (1.08–2.21)	**0.017**	1.19 (0.74–1.93)	0.176	0.470
Virus load (IU/mL)	1.24 (1.02–1.50)	**0.031**	1.18 (0.93–1.50)	0.166	0.170

*APTT* activated partial thromboplastin time, *SHR* sub-hazard ratio, *aSHR* adjusted SHR

### Prognostic factors analysis based on Fine–Gray test

**Fig. 2:**
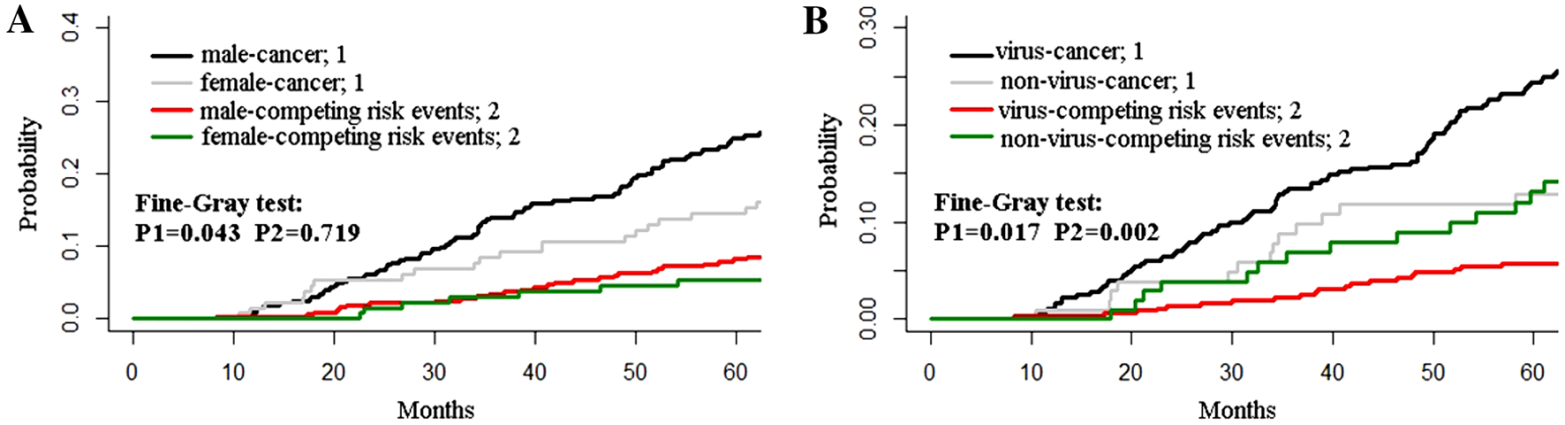
Cumulative incidence curves of hepatocellular carcinoma occurrence based on gender and etiology. **a** For gender, **b** for etiology.

Cumulative incidence curves were plotted based on gender and etiology (Fig. 2). For gender, after the Fine–Gray test, it could be seen that the cancer risk was a statistical difference between the two groups (Fig. 2a). The cumulative incidences of 1, 3, and 5 years in males were 0.9%, 14.0%, and 24.9%, while 1.5%, 8.4%, and 14.6% in females (*P* = 0.043). There was no significant difference in the incidence of competing risk events between the two groups. As shown in Fig. 2b, the cumulative 1-, 3-, and 5-year cancer incidences were higher in patients with virus-associated cirrhosis than that with non-virus-associated cirrhosis (1.1%, 13.4, 24.4% versus 1.0%, 8.8%, 12.9%; *P* = 0.017). However, the corresponding incidences of competing risk events were higher in patients with non-virus-associated cirrhosis than that in the other group (0%, 6.9%, 13.1% versus 0.3%, 2.3%, 5.8%; *P* = 0.002).

**Fig. 3:**
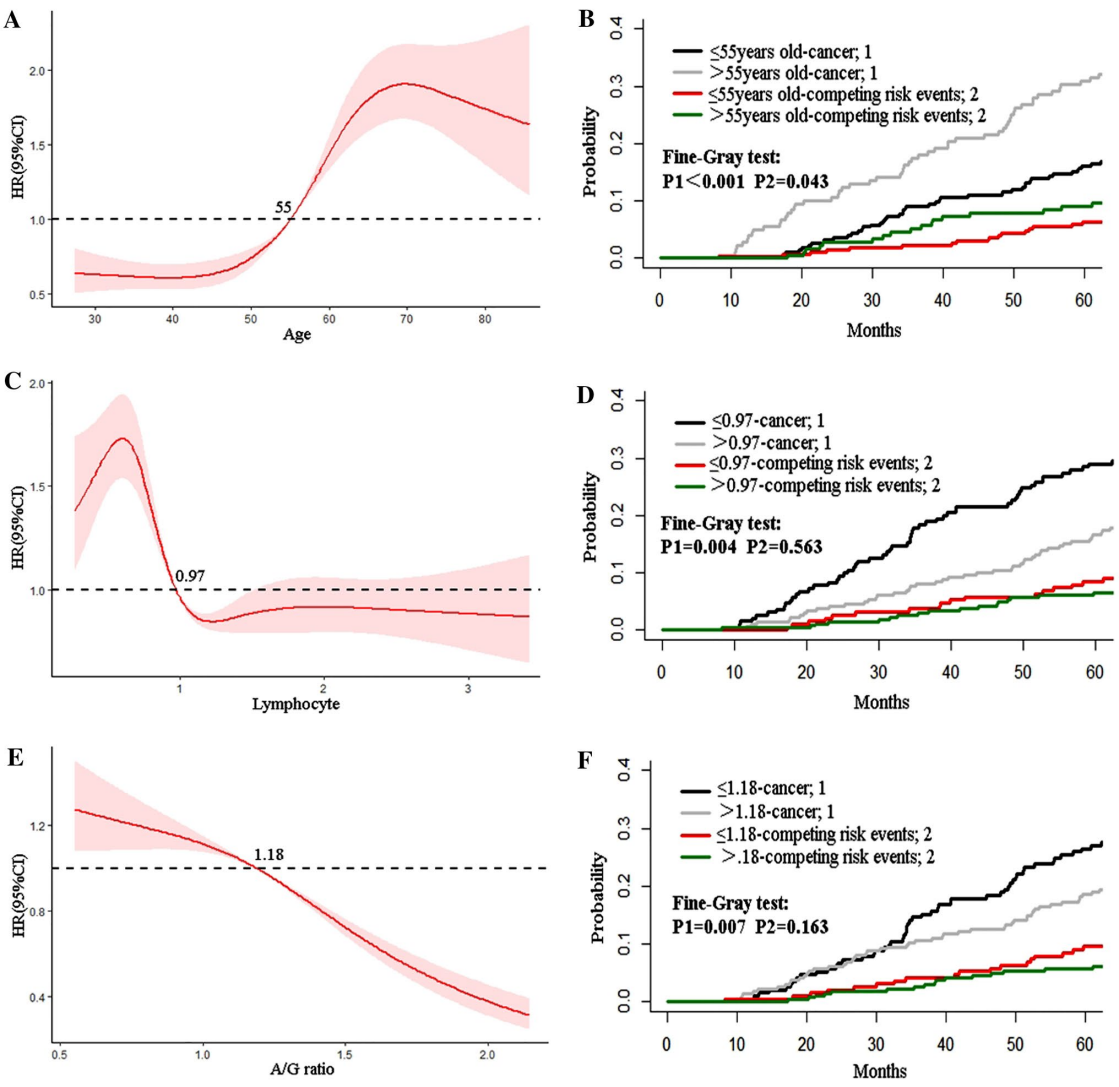
Analysis of non-linear relationships between predictors and HCC occurrence based on RCS and corresponding cumulative incidence curves. **a** and **b** Age; **c** and **d** lymphocyte; **e** and **f** globulin. *HCC* hepatocellular carcinoma, *RCS* restricted cubic splines

The non-linear relationship between the parameters of age, lymphocyte, and A/G ratio and cancer occurrence based on the RCS method was explored. The results showed that the risk of HCC tended to be stable before 55 years old and increased rapidly after that age (Fig. 3A). Therefore, the cutoff value of 55 was used to divide the patients into two groups and draw the cumulative incidence curves of both groups. Both the cumulative incidences of HCC and competing risk events were higher in patients older than 55 years old (Fig. 3b). The cumulative 1-, 3-, and 5-year cancer incidences were 0%, 9.1%, and 16.1% for patients under 55 years, while 2.8%, 17.5%, and 30.9% for patients over 55 years old, respectively (*P* < 0.001).

Similarly, we performed the above analysis on lymphocytes and the A/G ratio. The risk of cancer was significantly increased when the absolute value of lymphocyte was less than 0.97 (10^9/L) (Fig. 3C). Then the patients were divided into two groups with a cutoff value of 0.97, and it was found that there was a significant statistical difference in the cumulative 1-, 3-, and 5-year cancer incidences between the two groups (1.6%, 18.4%, 29.0% versus 0.8%, 8.0%, 16.7%; *P* = 0.004). There was no difference in the incidence of competing risk events (Fig. 3D). For the A/G ratio, the risk of cancer was significantly increased when it was less than 1.18 (Fig. 3E). The cutoff value of 1.18 was used to divide the patients into two groups, and we found that the cumulative 1-, 3-, and 5-year cancer incidences in low A/G ratio group were significantly higher than patients in high A/G ratio group (0.5%, 14.7%, 26.5% versus 1.5%, 10.7%, 18.5%; *P* = 0.007). There was no significant difference in the cumulative incidence of competing risk events between the two groups (Fig. 3F).

### Evaluation of discrimination and calibration of the established model

**Fig. 4:**
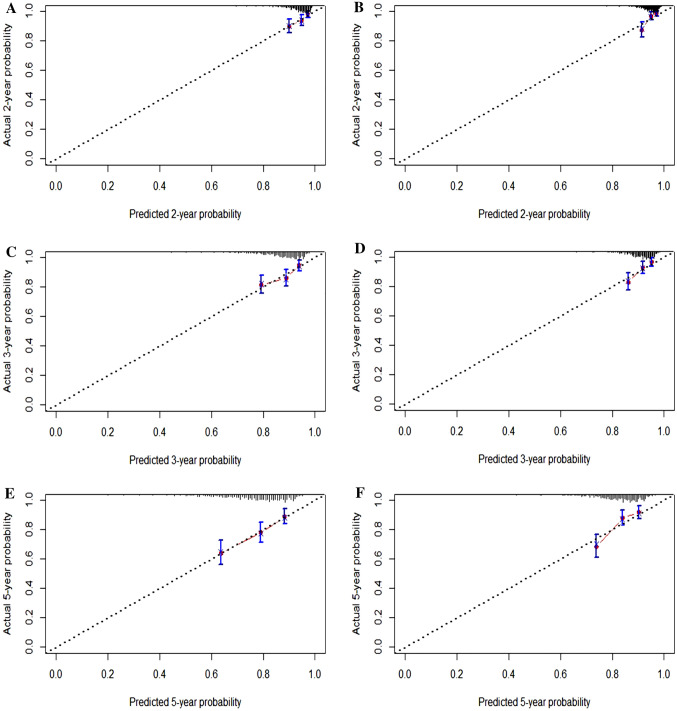
Calibration plots for predicting 2-,3-, and 5-year HCC occurrence in derivation cohort and validation cohort. (ACE) derivation cohort; (BDF) validation cohort. *HCC* hepatocellular carcinoma.

The model's discrimination (i.e., ability to distinguish those who will develop HCC from those who will not), calibration (i.e., the degree of consistency between the predicted probability by the model and the observed probability), and clinical value was assessed. The Harrell's concordance index of the model was calculated, with 0.732 for the derivation cohort and 0.729 for the validation cohort. Because the 1-year cumulative incidence of HCC was low, calibration curves of predicting 2-, 3-, and 5-year HCC occurrence were drawn in the two cohorts, respectively. It could be seen that the predicted probability was in good agreement with the observed probability (Fig. 4).

### Nomogram and analysis of clinical value

**Fig. 5:**
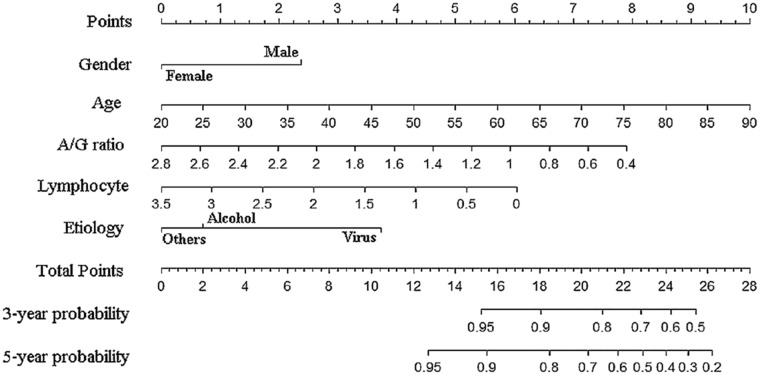
Nomogram used to predict time-dependent HCC occurrence in patients with cirrhosis. *HCC* hepatocellular carcinoma

**Fig. 6:**
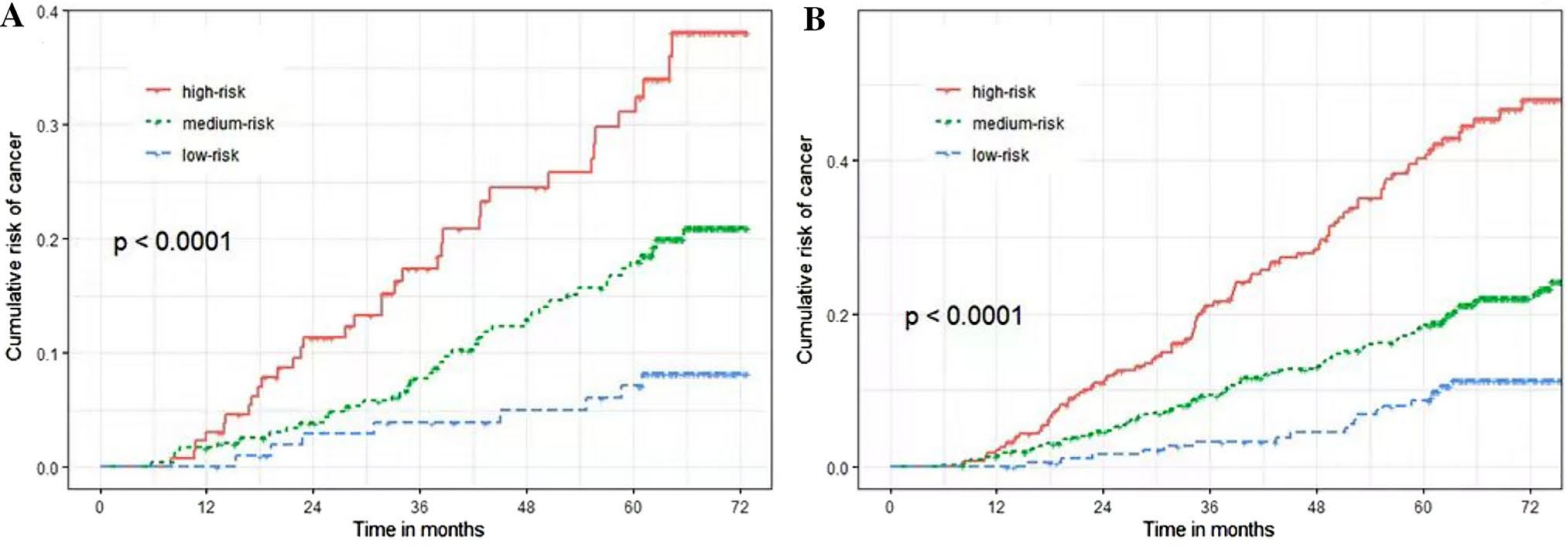
Cumulative incidence curves of HCC and competing risk events by tertiles of predicted 5-year risk (low-risk, medium-risk, and high-risk) in all patients. **a** HCC occurrence, **b** competing risk events

The nomogram based on the results of competing risk regression was plotted for clinical application (Fig. 5). For example, a 70-year-old male patient with alcohol-related cirrhosis, and with an A/G ratio of 1.2 and an absolute value of lymphocyte of 1.5 (10^9/L), had a total score of about 18.8, and the corresponding 3- and 5-year cancer incidences were about 12% and 22% (Table 4). To evaluate the model's ability to identify patients with different cancer risks, three groups (low-risk, medium-risk, and high-risk) were generated on account of tertiles of the 5-year risk predicted by the YOUAN model in the validation cohort and the whole cohort, and the cumulative incidence curves of the three groups were plotted, respectively. It was found that the YOUAN model could stratify patients in both the validation cohort and the whole cohort according to the disparate risk of HCC and competing risk events (Fig. 6). For the validation cohort, the cumulative 1-, 3-, and 5-year incidences of HCC were 3.5%, 17.5%, and 33.0% in the high-risk group, 2.0%, 8.9%, and 17.5% in the medium-risk group, and 0%, 4.8%, 7.3% in the low-risk group (*P* < 0.001). The corresponding incidences in the whole cohort were 2.0%, 20.8%, and 40.3% in high-risk group, 0.9%, 10.1%, and 19.7% in medium-risk group, and 0%, 2.0%, 9.5% in low-risk group (*P* < 0.001). In addition, patients with a higher risk of cancer had a higher risk of competing risk events, which meant that the YOUAN model could predict death to some extent (Supplementary Fig. 3).

**Table 4:** Predicted 3- and 5-year hepatocellular carcinoma occurrence risk of different patients using nomogram

Patients	1		2		3	
	Predictors	Points	Predictors	Points	Predictors	Points
Gender	Male	2.3	Male	2.3	Male	2.3
Etiology	Virus	3.8	Alcohol	0.7	Others	0
Age	75	7.8	70	7.1	65	6.4
A/G ratio	1.0	5.9	1.2	5.3	1.8	3.3
Lymphocyte	1.0	4.3	1.5	3.4	2.0	2.6
Total points	24.1		18.8		14.6	
3-year risk(1–*3-year probability*)	39%		12%		5%	
5-year risk(1–*5-year probability*)	60%		22%		9%

## Discussion

In this study, we successfully developed and validated a simple and accurate YOUAN model to predict the risk of HCC based on the competing risk regression, which contains five clinical indicators of routine examination, involving gender, age, etiology of cirrhosis, lymphocyte, and A/G ratio. The model showed excellent discrimination and calibration in assessing the cumulative cancer incidences of 2, 3, and 5 years in both the derivation cohort and validation cohort, regardless of etiologies of cirrhosis. The 5-year incidence of HCC could reach 40% in the high-risk group while less than 10% in the low-risk group. To date, the aMAP score was the first accurate, high-level, and simple-to-use model to predict individualized HCC risk for patients with chronic liver disease in the world, regardless of etiology, ethnicity or antiviral therapy (Fan et al. 2020). Likewise, we also developed the YOUAN model that stratifies patients according to the different risks of cancer, regardless of etiologies of cirrhosis. Therefore, in this study, the predictive performance of the YOUAN model was compared with aMAP score. The Harrell's concordance indexes of the YOUAN model were higher than that of aMAP score in both the derivation cohort (0.732 versus 0.692) and the validation cohort (0.729 versus 0.705).

We found that HCC occurrence was the highest in patients with virus-associated cirrhosis, with more than 50% in patients with co-infection of HBV and HCV, while mortality was the highest in patients with cirrhosis of other causes with the lowest cancer incidence. Both males and patients aged older than 55 had a higher risk of cancer than females and younger patients. Other studies have also reported that age and gender were independent predictors for evaluating the occurrence of liver cancer (Yang et al. 2020; Kim et al. 2018; Papatheodoridis et al. 2016; Ioannou et al. 2018, 2019; Yu et al. 2019; Sharma et al. 2017). For the etiology of cirrhosis, chronic HBV or HCV infection was still the most important cause of liver cancer so far, and the annual incidence of HCC was 2–5% in patients with virus-related cirrhosis (El-Serag 2012; Yang et al. 2016; Yang and Roberts 2010). Alcoholic liver disease was the second most common risk factor for liver cancer (Park et al. 2015). Other chronic liver diseases, such as chronic biliary tract disease and hereditary or metabolic liver disease, could also lead to cirrhosis and further promote the development of cancer, but the proportion of cancer caused by these etiologies was less than 5% to 10% worldwide (Yang and Roberts 2010). The above reports were completely consistent with our studies.

Most notably, the YOUAN model involved two clinical indicators that had not been considered in other models, namely lymphocyte and A/G ratio. Lymphocyte, which played an important role in the immune response, was a major factor in inhibiting cancer progression. As a parameter reflecting the strength of the body's immunity, the reduced number of lymphocytes indicated that the body lacked an effective immune response to tumors (Li et al. 2017; Iseki et al. 2017). Previous studies have revealed the potential relationship between chronic inflammation and cancer and found that inflammatory mediators in cells, such as interleukin—6 (IL-6) and tumor necrosis factor-α (TNF-α), could change the tumor microenvironment and promote the proliferation, malignant transformation, and metastasis of tumor cells (Pfensig et al. 2016; Arroyo et al. 2014). The serum A/G ratio was one of the important markers to reflect systemic inflammation. On the one hand, albumin was related to the nutritional status of patients. Hypoproteinemia in patients meant malnutrition, decreased immunity, and weakened defense ability. On the other hand, inflammatory factors such as IL-6 and TNF-α could affect the synthesis of albumin by hepatocytes, thus increasing the risk of infection and promoting the invasion and metastasis of tumors (Gupta and Lis 2010). Studies have shown that low albumin led to immunosuppression, impaired lymphocyte function, and reduced lymphocyte count (Chen et al. 2015). High levels of globulin could be regarded as a marker of the activated inflammatory response (Macfarlane et al. 2016). The composition of globulin was more complex, including interleukin, C-reaction protein, etc., which play an important role in the occurrence, development, and metastasis of tumors.

There were some limitations to our study. First of all, this was a single-center study. However, we divided the patients into derivation cohort and validation cohort according to the time of visit. Temporal validation, as a type of external validation, could strengthen the transportability and generalization ability of the model. Second, the YOUAN model only included five clinical indicators of routine examination and did not take into account other variables (such as proteins or metabolites, and circulating cell-free DNA signatures). The original intention of this study was to develop an economical and cost-effective prediction model based on routine laboratory indicators for clinical application. Nevertheless, to further optimize the model established, our team will consider combining the above indicators with the existing model in future work. Third, the study was conducted based on an Asian population, limiting predictive power for patients of other races.

Our study had several advantages. First, indicators involved in this study covered a wide range, including 34 variables of demographic data, etiology of cirrhosis, blood routine examination, liver and renal function examinations, coagulation markers, and others. Second, a competing risk model with a consideration of competing risk events was performed. Focusing only on cancer occurrence and ignoring competing risk events would lead to biased estimates of individual incidence. Third, it was the first study to incorporate lymphocyte and the A/G ratio as predictors of HCC occurrence into the model. Some studies included albumin instead of the A/G ratio in their models (Kim et al. 2018; Ioannou et al. 2018, 2019; Yu et al. 2019). However, compared with albumin or globulin alone, the A/G ratio was not easily affected by changes in body fluids, such as hemoconcentration or hemodilution, which could be used as a more objective and stable clinical indicator to assess the risk of cancer in patients. Finally, the etiological profiles of cirrhosis and HCC developing from cirrhosis of different causes were delineated in as much detail as possible. And the YOUAN model was developed based on patients with all-cause cirrhosis, while most other models for a patient population with a specific etiology, such as virus-associated cirrhosis, alcoholic liver disease, non-alcoholic fatty liver disease, etc. (Papatheodoridis et al. 2016; Ioannou et al. 2018, 2019; Alexander et al. 2019). And the YOUAN model could also predict death to some extent.

As an approach to decrease the cost and increase the cost-effectiveness, early diagnosis through the development of a personalized HCC monitoring strategy was still the best solution to improve the possibility of curing liver cancer and reducing mortality. Our model could screen out patients with a low risk of cancer occurrence, who could receive less intensive liver cancer surveillance, thereby saving medical resources. In contrast, for patients with high cancer risk, enhanced follow-up or more accurate but expensive imaging techniques were recommended for screening and diagnosis of HCC.

In response to the ambitious goal of reducing hepatitis-related mortality by 65% by 2030 set by the World Health Organization (WHO) (Mbuagbaw et al. 2017), we developed the “YOUAN model” that stratifies patients according to the different risks of cancer, regardless of etiologies of cirrhosis, which would be an effective and operable tool to improve the early diagnosis of liver cancer and reduce the mortality.

### Supplementary Information

Below is the link to the electronic supplementary material.

Supplementary file1 (TIFF 15360 KB)

Supplementary file2 (TIFF 8257 KB)

Supplementary file3 (TIFF 5406 KB)

Supplementary file4 (DOCX 14 KB)

## Data Availability

The datasets used and/or analyzed during the current study are available from the corresponding author on reasonable request.
