# Supplementary figures and images for: Individual surveillance by competing risk model for patients with hepatocellular carcinoma occurrence in all-cause cirrhosis
Source: J Cancer Res Clin Oncol. 2023 Jul 26;149(14):13403–16. doi: 10.1007/s00432-023-04911-y (PMC10587216; doi:10.1007/s00432-023-04911-y)

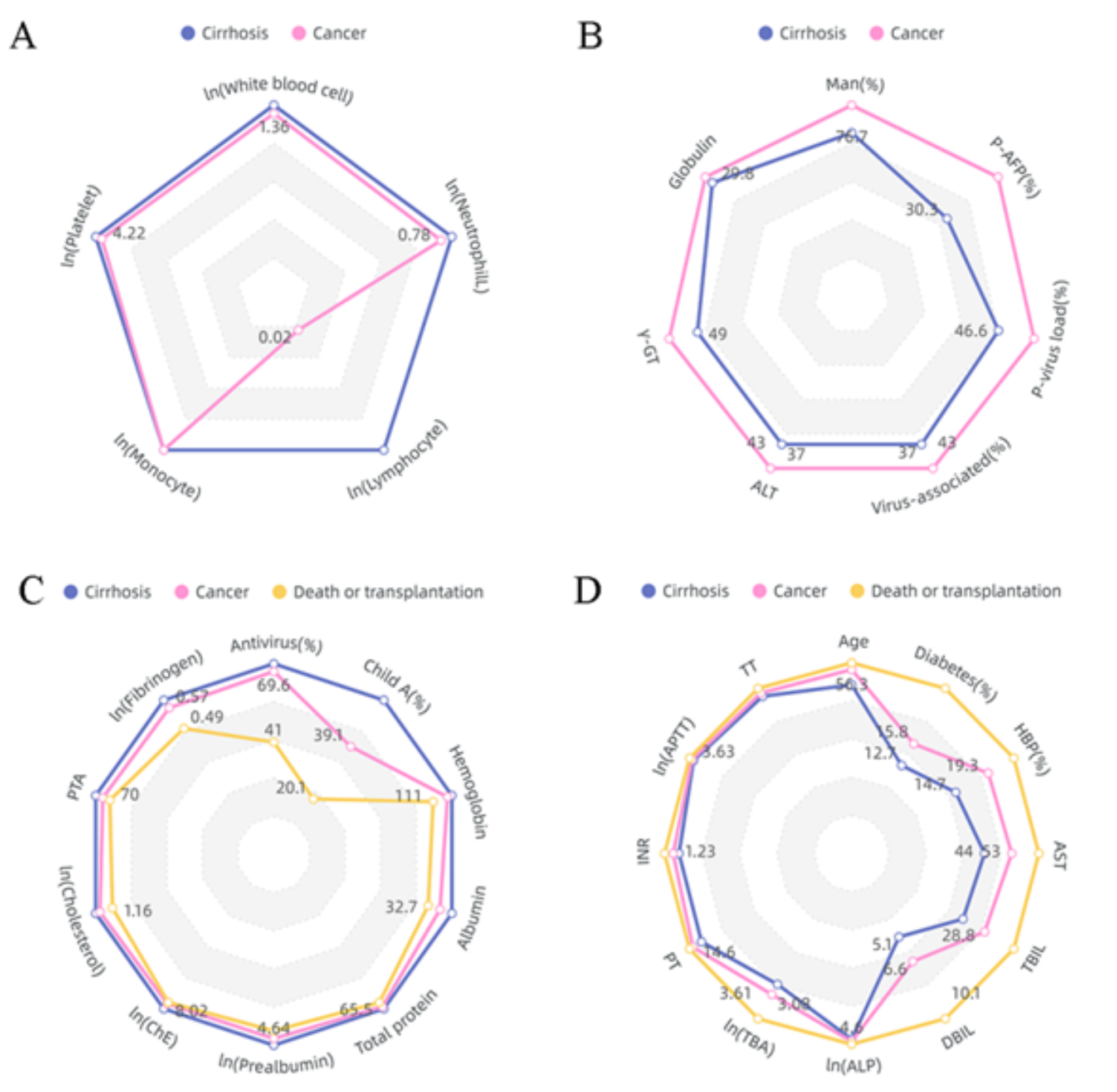

Supplement: Supplementary file 1 — Supplementary file1 (TIFF 15360 KB) [file 432_2023_4911_MOESM1_ESM.tiff]

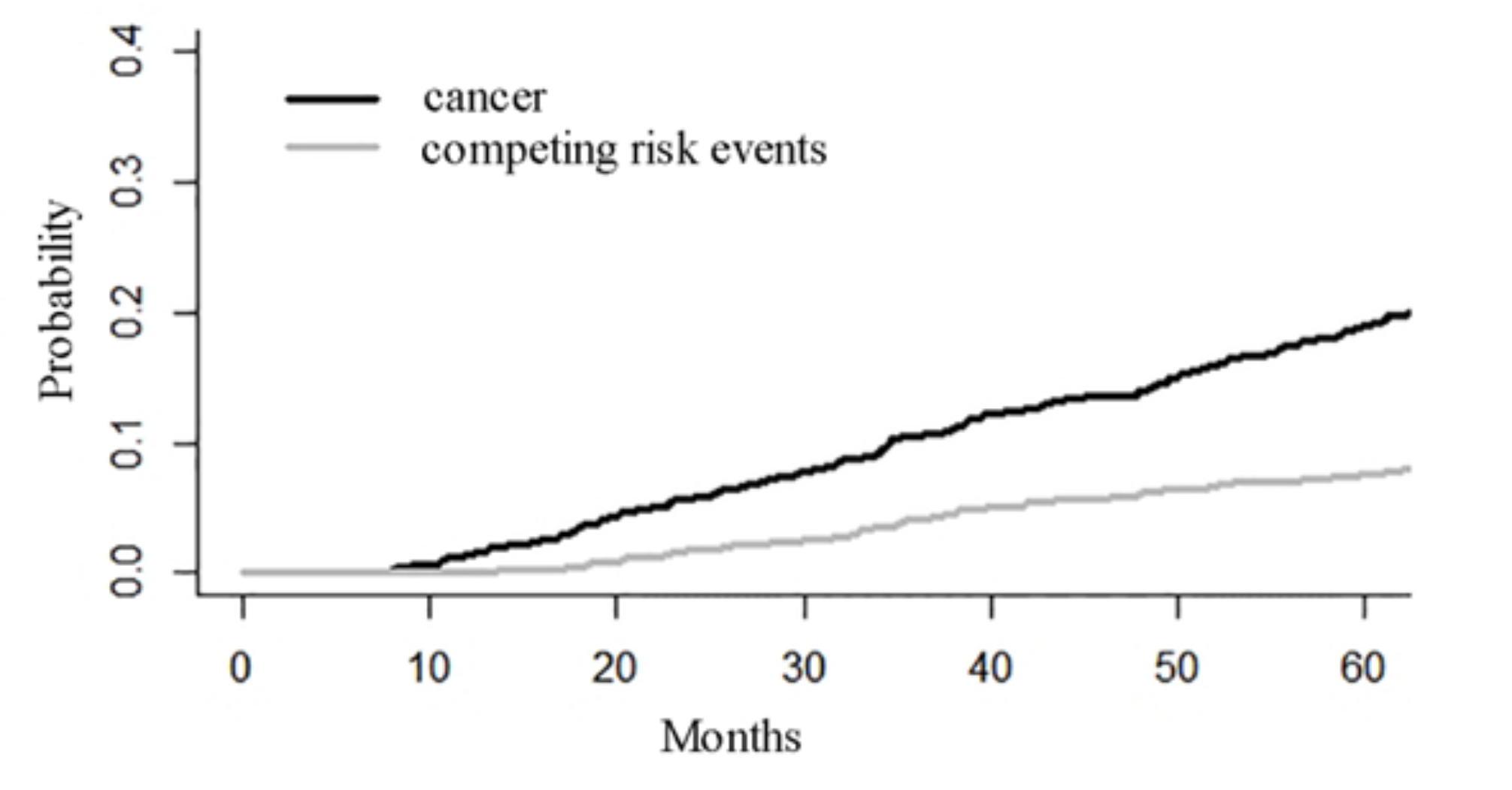

Supplement: Supplementary file 2 — Supplementary file2 (TIFF 8257 KB) [file 432_2023_4911_MOESM2_ESM.tiff]

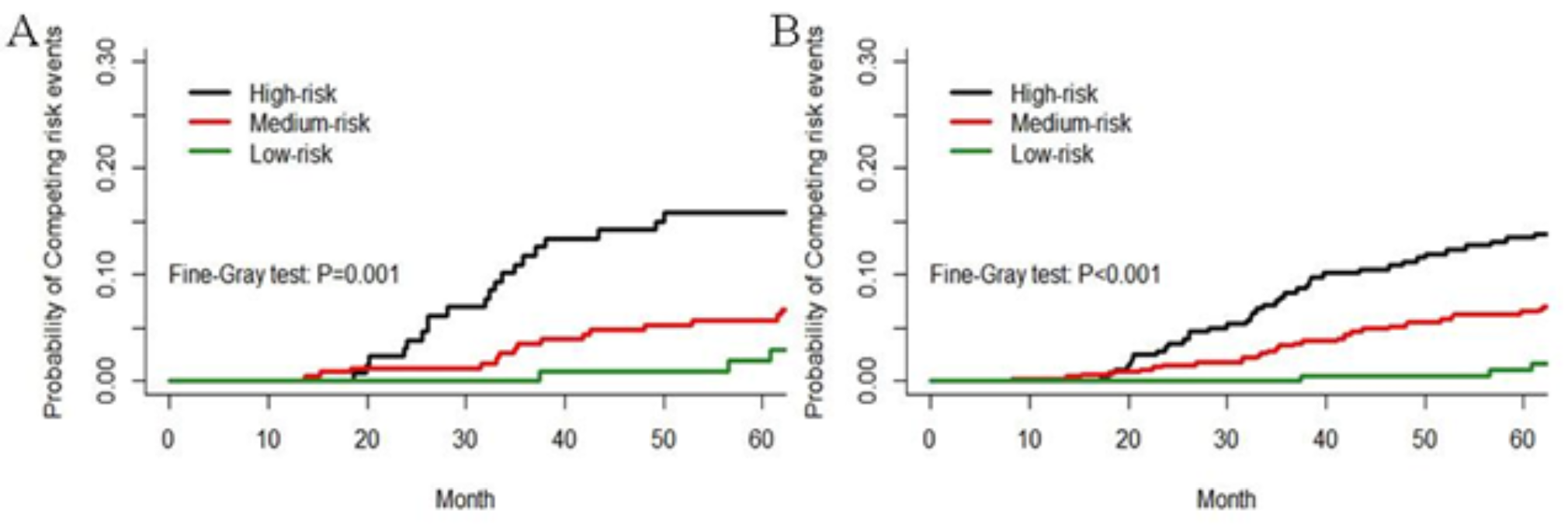

Supplement: Supplementary file 3 — Supplementary file3 (TIFF 5406 KB) [file 432_2023_4911_MOESM3_ESM.tiff]
