# Supplementary material for: Individual surveillance by competing risk model for patients with hepatocellular carcinoma occurrence in all-cause cirrhosis
Source: J Cancer Res Clin Oncol. 2023 Jul 26;149(14):13403–16. doi: 10.1007/s00432-023-04911-y (PMC10587216; doi:10.1007/s00432-023-04911-y)
Supplement: Supplementary file 4 — Supplementary file4 (DOCX 14 KB) [file 432_2023_4911_MOESM4_ESM.docx]

**Supplementary table 1**. Characteristics of hepatocellular carcinoma at diagnosis.

| **Variables** | **Number(n)** | **Percentage (%)** |
| --- | --- | --- |
| Tumor number (n) |  |  |
| 1 | 142 | 70.3% |
| 2 | 18 | 8.9% |
| ≥3 | 42 | 20.8% |
| Tumor size (mm) |  |  |
| ≤20 | 91 | 45.0% |
| 21-30 | 45 | 22.3% |
| 31-50 | 45 | 22.3% |
| ≥51 | 21 | 10.4% |
| Tumor stage (BCLC) |  |  |
| A | 134 | 66.3% |
| B | 25 | 12.4% |
| C | 16 | 7.9% |
| D | 27 | 13.4% |
| Terminal state (n) |  |  |
| Not | 175 | 86.6% |
| Vascular invasion | 16 | 7.9% |
| Distant metastasis | 11 | 5.5% |
